# Preoperative pain associated with rotator cuff tears correlates with synovitis severity in the rotator interval

**DOI:** 10.1016/j.jseint.2025.09.013

**Published:** 2025-10-24

**Authors:** Yuichi Nagase, Kazuki Abe, Kazuya Tamai, Masashi Naito, Hideaki Asai, Masahiko Morishige, Sakae Tanaka

**Affiliations:** a Department of Rheumatic Surgery, Tokyo Metropolitan Tama Medical Center, Tokyo, Japan; b Department of Orthopaedics, Tokyo Metropolitan Tama Medical Center, Tokyo, Japan; c Department of Orthopaedics, Tohto Bunkyo Hospital, Tokyo, Japan; d Department of Orthopaedics, National Hospital Organization Sagamihara Hospital, Sagamihara City, Kanagawa Prefecture, Japan; e Department of Orthopaedic Surgery, Yashio Central Hospital, Yashio City, Saitama Prefecture, Japan; f Department of Orthopaedic Surgery, Zenshin Orthopaedics Sports Rehabilitation Clinic Tachikawa, Tokyo, Japan; g Department of Orthopaedics, The University of Tokyo Hospital, Tokyo, Japan

**Keywords:** Rotator cuff tear, Pain, Synovitis, Rotator interval, Arthroscopic findings

## Abstract

**Background:**

The present study primarily aimed to investigate whether preoperative pain associated with rotator cuff tears correlates with the severity of synovitis in the rotator interval (RI) on arthroscopic findings retrospectively.

**Methods:**

Between July 2017 and December 2022, 131 shoulders with arthroscopic rotator cuff repair were retrospectively investigated. The pain domain of the preoperative Constant Score and Shoulder36 were used to assess preoperative pain. Two examiners confirmed the degree of synovitis of RI in the arthroscopic findings.

**Results:**

The mean patient age was 68.9 years. Synovitis in the RI was found in 118 shoulders (94%) assessed by arthroscopy. The pain domain of the Constant Score significantly correlated with synovitis in the RI assessed by arthroscopic findings (*P* < .01), but the size and the number of rotator cuff tears did not.

**Conclusion:**

The preoperative pain associated with rotator cuff tears significantly correlated with synovitis severity in the RI assessed by AS findings, highlighting the role of synovitis as a potential contributor to pain in patients with rotator cuff tears.

Rotator cuff tears, the most common cause of shoulder disability, have a prevalence of approximately 25%-50% in the elderly population.^13^^,^^21^ Many patients with rotator cuff tears undergo surgery due to shoulder pain, although the proper focus of pain remains unclear.

Previous studies have produced inconsistent results regarding the relationship between tear size and pain. Rizvi et al reported that smaller tear size, younger age, female sex, and work-related injuries were associated with greater postoperative pain after arthroscopic rotator cuff repair (ARCR), particularly at 6 weeks postoperatively.^14^ Another study also demonstrated that smaller tears were more painful than larger tears between 6 weeks and 6 months after surgery, although these data did not include preoperative pain.^22^ These findings suggest that healing processes may play a more critical role in postoperative pain, and smaller tears tend to be more painful during the healing process.

In terms of preoperative pain, a previous study reported that the pain severity related to rotator cuff tears was not associated with the size or number of the tears, but was associated with comorbidities, lower education level, and race.^6^ Moreover, approximately up to two-thirds of all rotator cuff tears were asymptomatic, and positive impingement sign, weakness of external rotation, and the presence of a tear in the dominant arm accounted for the symptoms associated with rotator cuff tears.^20^ In clinical practice, some symptomatic rotator cuff tears are successfully converted to asymptomatic tears without surgery.^20^ Elucidating the cause of the pain associated with rotator cuff tears may lead to a better understanding of the pathogenesis and treatment of the disease. Causes of shoulder pain include anatomical disorders, synovitis, bursitis, subacromial impingement, and lesions in the long head of the biceps brachii. However, the mechanism leading to the development of painful rotator cuff tears is poorly understood.

The rotator interval (RI) is an anatomical region in the anterosuperior aspect of the glenohumeral joint. It consists of a complex of fibers of the coracohumeral ligament, superior glenohumeral ligament, anterior edge of the supraspinatus, and superior edge of the subscapularis tendons. The synovium is a membrane lining the inner surface of the joint capsule. Inflammatory cytokines lead to lymphocytic infiltration, angiogenesis, and synovial thickening, which result in synovitis, a condition commonly observed during ARCR, especially in the RI.

Inflammatory processes in the shoulder may have been linked to pain. Abrams et al found that patients with full-thickness rotator cuff tears had greater synovial inflammation, angiogenesis, and matrix metalloproteinase 3 upregulation than control subjects. Moreover, the gene expression of matrix metalloproteinase 3 was found to correlate with the severity of synovitis.^1^ However, their study did not investigate the correlation between pain and the prevalence of synovitis. Stengaard et al demonstrated severe signs of acute inflammation, early degeneration, and fatty infiltration in the supraspinatus muscle after a rotator cuff tear in the C57BL/6 mice.^17^ Terabayashi et al reported increased anterior humeral circumflex artery blood flow in patients with night pain.^18^

Gotoh et al reported that interleukin-1 expression in the glenohumeral synovium contributed little to pain generation in rotator cuff disease,^7^ suggesting that the direct relationship between shoulder synovitis and pain remains unclear. In contrast, a systematic review has demonstrated a moderate association between synovitis and pain in knee osteoarthritis, indicating that joint-specific factors may underlie the heterogeneity of this relationship.^4^

Based on arthroscopic findings, Lee et al demonstrated that synovitis significantly correlated with the postoperative range of motion (ROM), strength, and Constant Score (CS) but not with the preoperative or postoperative visual analog scale (VAS).^12^ The VAS is widely used because of its simplicity but may lack reproducibility, whereas the CS pain score contains only 4 grades (none, mild, moderate, severe) and might have been more understandable to the patients.

Pain is a subjective parameter that can be influenced by numerous confounding factors, making it challenging to establish robust scientific evidence; nevertheless, it is one of the most important considerations during treatment. Reports on rotator cuff tears and synovitis have recently increased, suggesting a potential role of synovitis in pain generation. Candela et al reported that the macroscopic aspects of glenohumeral synovitis were related to the severity of rotator cuff tears. They emphasized the need to clarify the role of synovitis as a pain generator in future studies.^2^ Building on these findings, the present study specifically focuses on the correlation between preoperative shoulder pain in rotator cuff tears and the severity of synovitis observed intraoperatively, providing new insights into the role of synovitis as a pain generator.

Although a systematic review suggested a moderate association between knee pain and synovitis,^4^ no prior studies have specifically examined the correlation between preoperative pain and synovitis in the RI in rotator cuff tears assessed by arthroscopic findings.

Therefore, the present study aimed to investigate whether preoperative pain related to rotator cuff tears correlates with the synovitis severity in the RI on arthroscopic findings retrospectively.

## Materials and methods

### Patient selection

The present study retrospectively enrolled 141 patients with a degenerative or traumatic rotator cuff tear who underwent ARCR at Tokyo Metropolitan Tama Medical Center between July 2017 and December 2022. All cases underwent magnetic resonance imaging (MRI). The exclusion criteria were the presence of an inflammatory disease (eg, rheumatoid arthritis), neurodegenerative disease (e.g., Parkinson's disease), infection, and failure to complete a patient-based scoring system. All patients undergoing ARCR at our institution who met the eligibility criteria were enrolled consecutively during the study period. Because the study was conducted at a single tertiary referral center, it required approximately 5 years to collect an adequate number of cases for analysis. Enrollment was not based on voluntary participation; instead, all eligible cases during the specified period were included. Finally, 131 patients (68 males and 63 females) were enrolled. At least 3 months prior to surgery, no subacromial, bursal corticosteroid injection was administered preoperatively to any of the patients. The surgery was performed by 2 orthopedic surgeons familiar with ARCR.

### Collecting clinical data and applying the pain score

Preoperative pain was evaluated 1 month before surgery using the CS and the Shoulder36. The CS and Shoulder36 were collected at the same time point and by the same evaluator to ensure consistency. The CS is a 100-point, validated shoulder scoring system mainly used by clinicians to assess pain, function, ROM, and strength. Shoulder pain on the CS is categorized as none (15 points), mild (10 points), moderate (5 points), or severe (0 points).

Shoulder36 is a patient-reported outcome measure created by the Japanese Orthopaedic Association and the Japan Shoulder Society in 2011. It contains 36 questions in 6 domains (pain, ROM, muscle strength, general health, activity of daily living, and sports). The patients are asked to score 36 items using the following values: 0, unable to do it at all; 1, major difficulty and requiring help from someone; 2, some difficulty but able to manage on my own; 3, minor difficulty; and 4, no difficulty. The domain scores were the average of the scores for each domain. A previous study reported the correlation coefficient in each domain between Shoulder36 and Constant Score to be 0.62-0.74 in 230 patients with rotator cuff tears, demonstrating a significant association between the 2 scoring systems.^10^

The preoperative pain domain of the CS and Shoulder36 were retrospectively used to evaluate the degree of preoperative pain. The preoperative pain score was assessed for any correlation with age, sex, dominant side, tear size, tear site, number of tears, and intraoperative findings.

### Interpretation of the intraoperative findings

**Figure 1:**
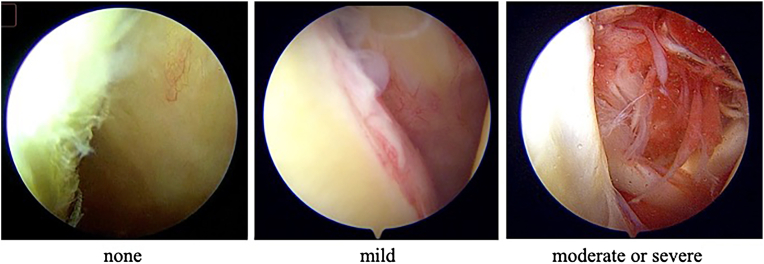
Score using arthroscopic findings to evaluate synovitis in the RI. Cases of synovitis in the RI were divided into 3 groups based on the following scores: 0 points (none), no reddish synovium and few, villous projections; 1 point (mild): slightly reddish synovium and a few, villous projections; 2 points (moderate to severe): reddish synovium and extensive, villous projections. *RI*, rotator interval.

The intraoperative findings of synovitis in the RI were classified following a previously reported method.^2^,^5^,^8^^,^^11^ Briefly, the degree of synovitis was scored as follows: The absence of reddish synovium and the presence of few, villous projections was scored 0 (none), the presence of slightly reddish synovium and a few, villous projections was scored 1 (mild), and the presence of reddish synovium and extensive, villous projections was scored 2 (moderate to severe), respectively (Fig. 1). Two experienced orthopedic surgeons also evaluated the intraoperative findings. When a discrepancy arose in grading between the 2 investigators, another coauthor reviewed the arthroscopic findings and determined which grade was appropriate.

### Definition of the size of rotator cuff tears

MRI was performed with MAGNETOM Skyra FIT (Siemens, Bavaria, Germany). Based on the preoperative MRI findings on either the oblique-coronal or oblique-sagittal views as well as the intraoperative findings, a partial tear was defined as a non–full-thickness tear; a small tear was defiled as a full-thickness tear less <1 cm in length; a medium-sized tear was defined as a full-thickness tear 1-3 cm in length; and a large tear was defined as a tear ≥3 cm in length according to the Cofield classification.^3^

### Statistical analysis

All the statistical analyses were performed using R (version 4.2.2; R Foundation for Statistical Computing, Vienna, Austria). All continuous variables were expressed as the mean ± standard deviation for parametric variables or the median (first-third quartile) for nonparametric variables. Categorical variables were expressed as numbers and percentages. Spearman's rank correlation coefficient was used to compare groups. In addition, the box plot was generated to visualize the distribution of pain intensity and synovitis severity. Moreover, to test for a trend in synovitis severity corresponding to increasing pain intensity, we performed the Jonckheere-Terpstra trend test statistic (JT), which is suitable for detecting ordered differences among groups when both variables are ordered. Pearson's chi-square test was used for 2 × 2 categorical data. The threshold for significance was *P* < .05.

This study was approved by the ethics committee of Tokyo Metropolitan Tama Medical Center (Approval No. 4-145).

## Results

The mean age was 68.9 ± 10.4 years, and 48% of the cohort (63 of 131 shoulders) consisted of females. The dominant side was affected in 69% of the cohort. Partial or small tears, medium-sized tears, and large-sized tears were found in 30.5%, 38.9%, and 30.5%, respectively (Table I). Synovitis of the RI was found in 94% of the cohort on arthroscopy (AS). Spearman's rank correlation coefficient between the investigators was 0.956. Most cases of pain were mild on the preoperative CS pain score (mean: 5.4) or 2.7 on the preoperative Shoulder36 pain score (full score: 4) (Table II).

**Table I:** Basic characteristics of the patients at arthroscopic rotator cuff repair.

Variables	All (N = 131)
Mean age (yr) (±SD)	68.9 (10.4)
Sex, female	63 (48.1%)
Dominant side (right)	91 (69.5%)
Affected side (right)	89 (67.9%)
Size of tears	
Small	40 (30.5%)
Medium	51 (38.9%)
Large	40 (30.5%)
Site of tears	
SSC	44 (33.6%)
SSP	125 (95.4%)
ISP	81 (61.8%)
Tm	1 (0.8%)
Number of tears	
1	39 (29.8%)
2	65 (49.6%)
3	26 (19.8%)
4	1 (0.8%)

*SD*, standard deviation; *SSP*, supraspinatus; *ISP*, infraspinatus; *Tm*, teres minor; *SSC*, subscapularis.

**Table II:** Preoperative arthroscopic findings and pain severity.

Variables	All (N = 131)
Synovitis in RI with arthroscopy	
None	8 (6.3%)
Mild	63 (50%)
Moderate or severe	55 (43.7%)
Preoperative CS pain	
Mean	5.4
None (0 points)	27 (20.8%)
Mild (5 points)	69 (53.1%)
Moderate (10 points)	31 (23.8%)
Severe (15 points)	3 (2.3%)
Preoperative Sh36 pain	
Mean	2.7 (1.0)

*RI*, rotator interval; *CS*, Constant Score; *Sh36*, Shoulder36.

**Figure 2:**
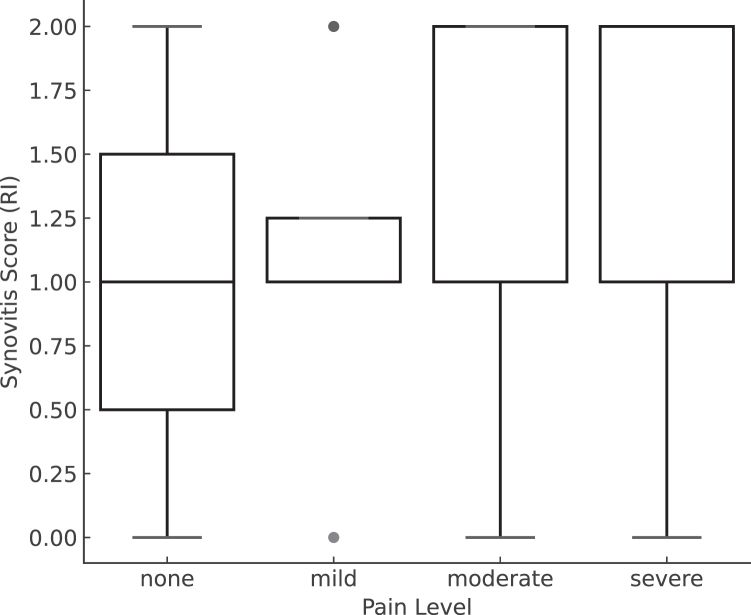
Boxplot of synovitis scores in the RI across 44 categories of preoperative pain levels. Pain levels were categorized based on the CS pain score: none, mild, moderate, and severe. Synovitis was arthroscopically graded as 0 (none), 1 (mild), or 2 (moderate to severe). The figure showed the distribution of synovitis severity across pain groups. *CS*, Constant Score; *RI*, rotator interval.

The CS pain score significantly correlated with synovitis in the RI assessed by AS images (Spearman's rank correlation test; *P* < .01) (Table III). The boxplot demonstrated a tendency for higher synovitis severity to be observed in groups with more pain (Fig. 2). The JT revealed a statistically significant positive trend in synovitis severity with increasing pain levels (JT = 2985.0, Z = 3.25, *P* = .001), indicating that patients with greater preoperative shoulder pain exhibited more synovitis severity in the RI. In addition, the pain score of Shoulder36 was correlated with synovitis severity (*P* = .04) with Spearman's rank correlation coefficient.

**Table III:** Correlation between the CS pain score and synovitis in the RI with AS.

Synovitis in the RI with AS	CS pain score				*P* value
	severe (0)	moderate (5)	mild (10)	none (15)	
None	1	4	2	1	<.01
Mild	7	35	19	1	
Moderate or severe	18	29	7	1	

*AS*, arthroscopy; *RI*, rotator interval; *CS*, Constant Score.

The numbers in the () indicate the score. Spearman's rank correlation test was used for the analysis.

The CS and Shoulder36 pain scores did not correlate with the size of the rotator cuff tear, number of tears, dominant side. In addition, the Shoulder36 pain score correlated with female sex (2-sample t-test; *P* < .001) while the CS pain score did not (Table IV). The correlation between sex and synovitis severity was tested using Pearson's Chi-square test and Spearman's rank correlation test, but neither result was statistically significant.

**Table IV:** Correlation between each pain score and other factors in patients with rotator cuff tears.

	CS pain score		Sh36 pain score	
Factors	Rho	*P* value	Rho	*P* value
Rotator cuff tear size	−0.081	.361	−0.159	.078
Rotator cuff tear number	0.001	.993	−0.118	.192
Dominant-side tears	−0.031	.724	−0.024	.79
Sex (1 female, 0 males)	0.05	.572	0.351	<.001

*CS*, Constant Score; *Sh36*, Shoulder36.

## Discussion

While prior research has primarily focused on the correlation between the severity of glenohumeral synovitis and the size of rotator cuff tears,^2^ our study highlights the shoulder pain and the synovitis severity localized to the RI. In the present study, we found that higher preoperative pain levels were associated with synovitis severity in the RI, as demonstrated in Fig. 2. Specifically, patients reporting moderate to severe pain exhibited higher synovitis severity compared to those with no or mild pain. Our findings are consistent with previous studies reporting an association between pain and synovial inflammation in rotator cuff disease or frozen shoulder assessed by MRI.^15^^,^^16^ On the other hand, a previous study demonstrated that glenohumeral synovitis evaluated by AS findings did not appear to have any correlation with preoperative pain or postoperative function. The author discussed 1 possible reason why the sample was underpowered for statistical analysis.^9^

The prevalence of synovitis in our series was higher than previously reported. This may be partly explained by the older mean age of our cohort, as some female patients with severe cuff tears underwent ARCR instead of arthroplasty as the initial surgical option. The older age profile may have contributed to the increased frequency of synovitis observed during AS.

Lee et al found that postoperative ROM and the clinical outcomes correlated with intraoperative synovitis, but that pre- and postoperative VAS scores did not correlate with synovitis in the superoanterior area of the joint capsule.^12^ The disadvantage of VAS is the lack of reproducibility. We are currently asking medical staff who are not directly involved in the treatment to measure VAS before and after surgery for future studies; however, VAS was not measured before and after surgery in all ARCR cases in the current study. One aspect of the present study in demonstrating the correlation between preoperative pain and synovitis in the RI may be attributable to the use of the CS pain score because of its simple 4-graded categories. However, it must be noted that this score reflects only one dimension of shoulder pain, although the CS pain score showed a trend consistent with increased synovitis severity. The CS pain score is limited in detail compared to other multidimensional pain assessment methods; therefore, the results of this study should be interpreted with caution. Hence, when evaluating pain, it is necessary to consider feelings of anxiety and depression, and it may be required to incorporate EuroQol 5-dimension 3-level questionnaire evaluations. Future studies incorporating multiple pain metrics may better elucidate the relationship between shoulder pain and synovitis severity.

Only a few studies have hitherto assessed a correlation between the pain associated with rotator cuff tears and inflammation, despite ample research investigating the functional disorders in patients with these conditions. Pain, which is often the main complaint of patients, is generally thought to be caused by inflammation or angiogenesis. Terabayashi et al reported that the peak systolic velocity of the anterior humeral circumflex artery in patients with a rotator cuff tear and night pain was higher than that of the unaffected side using pulse Doppler ultrasonography.^18^ The anterior humeral circumflex artery supplies blood flow to the RI; consequently, increased blood flow may promote angiogenesis in the RI, potentially leading to synovitis and night pain. For this reason, synovitis in the RI is usually coagulated using a radiofrequency device during AS to reduce postoperative pain.

In the present study, the CS pain scores did not correlate with tear size and number of tears. This contrasts with some previous studies that have reported smaller tears to be associated with greater pain severity.^14^^,^^22^ However, these data were on postoperative pain, and the data of the current study were on preoperative pain. That may be the reason for the discrepancy between the current data and data from previous reports. Another possible explanation is that pain in rotator cuff tears is multifactorial. Psychosocial factors such as mental health status, which were not assessed in our study, can also influence pain levels.^19^ On the other hand, our results are consistent with other report,^6^ suggesting that tear size alone is not a reliable predictor of pain, and evaluating inflammatory conditions and patient-related factors may be important when assessing shoulder pain.

It should also be noted that almost all patients in this study presented with some degree of synovitis in the RI assessed by AS, which may reflect an inclusion bias. Because all patients in this series underwent surgery for symptomatic rotator cuff tears, the cohort inherently consisted of individuals experiencing shoulder pain—one of the main surgical indications. However, synovitis severity varied (mild in 50% of cases and moderate to severe in 43.7%), and our additional analysis using both boxplot visualization and the Jonckheere–Terpstra trend test demonstrated that greater preoperative shoulder pain was associated with more severe synovitis in the RI.

The limitations of the present study are (1) its monocentric, retrospective design; (2) inadequate analysis of the patient background; (3) the possibility of interobserver disagreement in the interpretation of the intraoperative findings; (4) inclusion bias, such as age, grade of pain, and size of rotator cuff tear, because of the retrospective study; (5) the inclusion duration for the current study was relatively long, owing to the monocentric design; and (6) the lack of pain evaluation by VAS. To reduce potential bias, VAS should ideally be measured by medical staff who are not directly involved in patient care. In the present study, VAS was not obtained preoperatively and postoperatively in all ARCR cases; therefore, future studies should incorporate this standardized assessment. Another limitation of this study is that MRI data were excluded from the final analysis. Although MRI can provide noninvasive information about synovial changes, we considered arthroscopic assessment of synovitis to be more objective and reproducible. Despite these limitations, the present study found that the pain associated with rotator cuff tears correlated with synovitis in the RI, and these data may provide new insights into shoulder pain and synovitis.

## Conclusion

The preoperative pain associated with rotator cuff tears significantly correlated with synovitis severity in the RI assessed by AS findings. These findings highlight the importance of synovitis as a potential contributor to preoperative pain in patients with rotator cuff tears.

## Disclaimers:

Funding: No funding was disclosed by the authors.

Conflicts of interest: The authors, their immediate families, and any research foundation with which they are affiliated have not received any financial payments or other benefits from any commercial entity related to the subject of this article.
